# Forecasting urban water demand using different hybrid-based metaheuristic algorithms’ inspire for extracting artificial neural network hyperparameters

**DOI:** 10.1038/s41598-024-73002-w

**Published:** 2024-10-14

**Authors:** Salah L. Zubaidi, Hussein Al-Bugharbee, Ali W. Alattabi, Hussein Mohammed Ridha, Khalid Hashim, Nadhir Al-Ansari, Zaher Mundher Yaseen

**Affiliations:** 1 Department of Civil Engineering, Wasit University, Wasit, 52001 Iraq; 2 College of Engineering, University of Warith Al-Anbiyaa, Karbala, 56001 Iraq; 3 Department of Mechanical Engineering, Wasit University, Wasit, 52001 Iraq; 4 Advanced Lightning, Power and Energy Research (ALPER), Department of Electrical and Electronics Engineering, Faculty of Engineering, Universiti Putra Malaysia, 43400 Serdang, Malaysia; 5 Department of Computer Engineering, Mustansiriyah University, Baghdad, Iraq; 6 Department of Environmental Engineering, University of Babylon, Al‑Hillah, 51001 Iraq; 7 School of Civil Engineering and Built Environment, Liverpool John Moores University, Liverpool, UK; 8 Department of Civil Environmental and Natural Resources Engineering, Lulea University of Technology, 971 87 Lulea, Sweden; 9 Civil and Environmental Engineering Department, King Fahd University of Petroleum & Minerals, 31261 Dhahran, Saudi Arabia

**Keywords:** Urban water management, ANN, PSOGA, Metaheuristic algorithms, Water demand prediction, Environmental sciences, Engineering

## Abstract

This research offers a novel methodology for quantifying water needs by assessing weather variables, applying a combination of data preprocessing approaches, and an artificial neural network (ANN) that integrates using a genetic algorithm enabled particle swarm optimisation (PSOGA) algorithm. The PSOGA performance was compared with different hybrid-based metaheuristic algorithms’ behaviour, modified PSO, and PSO as benchmarking techniques. Based on the findings, it is possible to enhance the standard of initial data and select optimal predictions that drive urban water demand through effective data processing. Each model performed adequately in simulating the fundamental dynamics of monthly urban water demand as it relates to meteorological variables, proving that they were all successful. Statistical fitness measures showed that PSOGA-ANN outperformed competing algorithms.

## Introduction

Freshwater resources are extremely important and play a vital role in developing cities. The logical design and active management of the municipal water supply framework are of great importance to guarantee social and economic development^1^. Urban water demand is rising in many nations worldwide due to the escalating severity of climate change, population expansion, and economic development^2,3^. Water shortage has been well documented in developed and developing countries, leading to an imbalance between supply and requests for water^4,5^.

To properly manage modern freshwater resources, sound estimates and predictions of water demands in a certain area are essential to overcome the issue of water scarcity. This should be achieved because various cities in the United States (US), particularly those in arid regions such as the state of Texas in the southwest of the US, encounter problems of water scarcity. As such, authorities and engineers are continually trying to keep up with the rising demand resulting from the growing impact of socio-economic, political, and weather factors^6^. DeMaagd and Roberts^7^ stated that shifting patterns of rainfall are anticipated to influence surface water and aquifers worldwide, some of which, such as the Southwestern US, are already increasingly strained. Reliable estimates of water requests that account for factors driving consumption are essential to understanding future municipal water demands^6^.

Additionally, short-term water demand forecasting aids in administering and operating water supply systems. As an illustration, forecasts of short-term demand help water managers balance the needs of water supply to make better-informed decisions regarding water management. Reliable urban water demand forecasting models must be established in order to help ensure reliable water availability and reduce peak water use. These forecasting models help water utilities make tactical and strategic decisions, enhancing water security and the sustainability of water use^8^.

Water demand datasets typically exhibit non-stationary and non-linear behaviour at various spatial and temporal dimensions^9^. With the development of machine learning (ML) models, different versions of ML models were adopted to solve the associated non-linear nature of water demand variability. Some examples of these models are artificial neural networks (ANNs)^10^, support vector machines^11^, random forests^12^, gene expression programming^13^, long short-term memory neural networks^14^, and fuzzy logic^15^.

An analysis of different methods for predicting urban water demand over the last several decades^16–20^ detected that the ANN model was used effectively for different scenarios. ANN techniques have revealed optimistic progress in estimating short-, medium-, and long-term municipal water requests. Also, among several ML models, ANN showed a predominant model applied for various hydrology fields^21,22^. However, the capacity of individual ML methods to understand intricate data patterns and relationships is typically restricted, especially when dealing with non-linear and high-dimensional data^23^. This may lead to less efficient and accurate results when contrasted with more advanced ML models^24^. To tackle these limitations, an optimising method was developed.

Metaheuristic algorithms (MHAs) show promise in solving a variety of challenging non-linear issues across hydrological disciplines when comparing hybrid models to single ML models, such as^25–28^. These combined techniques are preferable to solving complicated real-world problems^29^. This is why these methods have shown improvement in identifying new regions that could lead to a broader set of solutions^30^. Not only that, but the ability to exploit is boosted so that local minima are avoided. Finally, when dealing with complex, multi-variable, and non-linear problems, these methods perform better^31^. Regardless, optimisation is still necessary, especially in forecasting hydrological factors due to their stochastic, data noise, and non-stationary nature^32^.

Exploration and exploitation are the two main parts of MHAs. In computer science, “exploration” refers to the steps used to identify the boundaries of a search space for an algorithm, while “exploitation” delineates the process of picking the best option out of a multitude of generated possibilities^33^. Finding the optimal balance between exploring and exploiting is essential for a search algorithm’s performance^34^. The relationship between exploration and exploitation, however, is inversely proportional^35^. MHAs can be classified based on their inspired technique into physics (P), such as gravitational search algorithm (GSA), evolutionary algorithms (EA) like genetic algorithm (GA), and swarm intelligence (SI), for example, particle swarm optimisation (PSO) and grey wolf optimiser (GWO)^36^. The effectiveness of improving algorithms to increase their efficiency in tackling optimising issues, either by altering existing algorithms (single-based) or combining different algorithms into hybrid ones (hybrid-based)^25^.

The framework of the suggested research methodology depends on data preprocessing techniques (i.e., normalisation, cleaning, and selecting best predictors) and prediction models^37^. Data preprocessing techniques are crucial steps that help to reduce the multicollinearity between predictors, improve data quality, and remove redundant variables, resulting in an improved forecasting model performance^38^. However, previous research studies of hydrological hybrid models have suffered from notable methodological weaknesses, leading to decreased prediction range and increased uncertainty. Considering data preprocessing techniques, previous studies have not dealt with one or more steps of data preprocessing methods, such as normalisation data^39,40^, cleaning data^41,42^, and selecting the best predictors^43,44^ or unimplemented all these steps^45,46^. Also, along with this growth in hydrological hybrid models, however, there are certain drawbacks associated with using hybrid models of prediction in multiple previous studies. One major drawback of these studies is that they hybridised the ML model with one MHA^47,48^. Hybridised the ML model with MHAs, which have the same inspiration^49,50^, is another (potential) limitation. Another problem with these studies is that they hybridised the ML model with only single-based MHAs^51,52^. Additionally, these studies, however, suffer from the fact that they were applying each swarm of MHA with few iterations^32,53^. Finally, applying each swarm of MHA one time^54,55^ is another potential concern.

Considering all of that, this study will compare the performance of the different hybrid MHAs based on their inspiration, and no literature has compared these MHAs before in this sector. It includes PSOGA (SI combined with EA), CPSOCSA (SI combined with P), PSOGWO (SI combined with SI), the modified PSO (MPSO), and PSO algorithm (as a benchmarking model) to forecast urban water demand. The above MHAs were applied effectively in different hydrology fields, such as PSOGA^56,57^, CPSOCSA^58,59^, PSOGWO^60^, MPSO^54,61^, and PSO^62^.

Water demand forecasts have lately become a very active area of research, as they lead to considerable environmental and economic benefits. A precise water demand forecast ensures a dependable water distribution system that is capable of providing users with potable water in sufficient volumes and adequate pressure^63^. The field of water demand estimation has become more critical, resulting from water resource shortages and a growth in water usage. Consequently, real doubts persist among water utility decision-makers regarding the present urban water system’s capacity to handle the exponential increase in water demands^64^. Therefore, the present study aims to address the following research objectives in light of the limitations highlighted in the literature review:

1. The capacity of the wavelet transformation (with various mother wavelets and orders) was adopted for data pre-processing as an advanced stage for the prediction process.
2. A new combined ANN-PSOGA model was developed based on the combination of the ANN model and hybrid nature-inspired algorithms for water demand prediction using ten years dataset belonging to College Station City, USA.
3. The proposed combined model ANN-PSOGA was validated against several other combined models (ANN-CPSOCGSA, ANN-PSOGWO, ANN-MPSO, and ANN-PSO) for validation and benchmarking purposes.
4. For each population size, the forecasting range was increased, and the uncertainty was decreased by repeating the process five times.

The remainder of this paper has been divided as follows. In “Study area and data set” section deals with the study area and data set. In “Methodology” section explains the methodology. Results are covered in “Results” section. In “Discussion” section considers the discussion. In “Conclusion” section presents the conclusion.

## Study area and data set

The present research adopted a catchment area in the USA located in College Station, TX, to build and evaluate the water demand model. The water services for the approximately 150,000 residents of this area are provided by the city’s municipal government. The region that is serviced is around 140 km^2^, with a residential service population of more than 95,000 customers and around 55,000 for commercial and industrial purposes. Water is supplied by the City’s nine groundwater wells and conveyed to the Dowling Road Pump Station by the Sandy Point Pump Station and transmission lines. Table 1 provides the statistical indicators of the dependent and independents data, including the urban water usage (megalitre, ML), maximum temperature (Tmax) (^o^C), minimum temperature (Tmin) (^o^C), mean temperature (Tmean) (^o^C), rainfall (Rain) (mm), solar radiation (Srad) (MJ/m^2^), maximum relative humidity (RHmax) (%), and wind (m/s) from January 2005 to July 2014.

**Table 1:** Statistical indicators of the collected data.

Variable	Maximum	Minimum	Mean
Water consumption (ML)	715,798,498	207,601,706	366,849,231
Tmax (^o^C)	41.2	11.4	26.8
Tmin (^o^C)	26.3	2.6	14.8
Tmean (^o^C)	33.8	7.1	20.8
Rain (mm)	326.8	2.1	75.1
Srad (MJ/m^2^)	28.3	17.1	6.7
RHmax (%)	0.83	0.35	0.64
Wind (m/s)	4.7	2.1	3.1

## Methodology

**Fig. 1:**
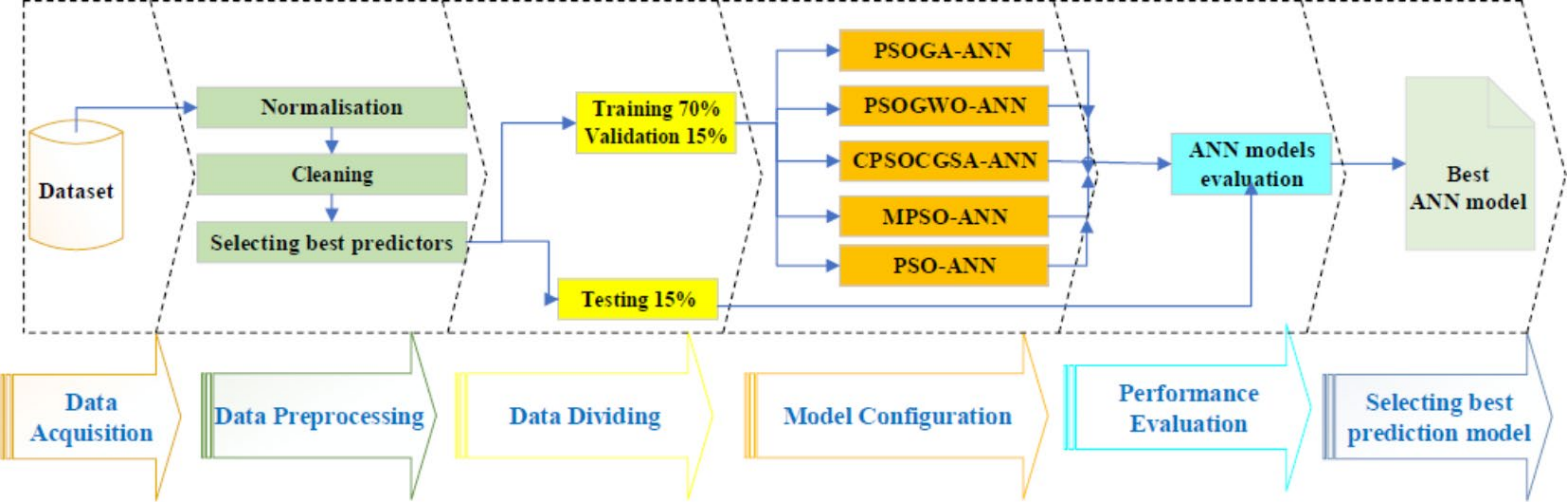
Framework of the suggested research methodology.

This research suggests a novel methodology focused on understanding the forecast monthly consumption of urban water based on weather variables (see Fig. 1). The framework of the suggested research methodology started with data acquisition from College Station, USA, (“Study area and data set” section). This is followed by data preprocessing techniques that include three steps responsible for enhancing raw data quality and selecting the best set of predictors. The data was then divided into three sets (i.e., data dividing): training, validation, and testing. Afterwards, five MHAs were combined with ANN to determine the optimal hyperparameters for the ANN model (i.e., model configuration stage). Next, the performance evaluation was conducted. In this stage, all the models are conducted. Finally, all the models are compared based on different statistical and graphical tests to select the best prediction models. The methodology will be described in the subsequent sections (i.e., from 3.1 to 3.6).

### Data preprocessing

It is a valuable technique for the prediction model, and it is categorised into normalisation data, cleaning data, and determining the optimum predictors^28^. Time series normalisation is best accomplished using the natural logarithm since it mitigates the impact of outliers and eliminates collinearity across predictors^37,65^. The cleaning of data can be implemented by applying the box and whisker method to determine and treat the outliers’ data. Then, denoising the time series after that by wavelet transform (WT) technique.

WT is a popular time–frequency analysis method. The principles of WT contain shifting and scaling the so-called mother wavelet along with the original time series to obtain a time–frequency representation. This WT can also be utilised to denoise the original time series. The principles of wavelet denoising contain localising time series information at different scales. Then, the important information (i.e., large-magnitude wavelet coefficients) is preserved while the noise (i.e., small-magnitude wavelet coefficients) can be shrunk or removed using thresholding. The denoised time series can be reconstructed using inverse wavelet transform^66,67^. Various mother wavelets are utilised and investigated in this study, and the most effective one in terms of providing a higher correlation between dependent and independent parameters is selected. The process of denoising and reconstructing can be conducted using the MATLAB toolbox.

The final step is to locate the optimum scenario of predictors, and for this reason, seven independent variables (weather variables) were determined as potential predictors of monthly urban water demand. The covariates were screened for inclusion in the prediction models utilising the tolerance technique to account for a high level of collinearity within the potential independent variables. Covariates with a tolerance coefficient of less than 0.2 were removed because they make little to no enhancement in forecast quality^65^.

### Genetic algorithm enabled particle swarm optimisation

The property of intensification provided by the genetic algorithm (GA) and diversification of PSO are combined to provide a better solution^68^.

The first step in PSO is to propose an initial population representing the potential solution. The individuals (i.e. particles) of this population are represented by the Eq. (1)

1 $$X_{i} = \left( {X_{i1} , X_{i2} , X_{i3} , \ldots \ldots ,X_{iD} } \right)$$

where *i* is the particle number, and *D* is the dimension. As in birds swarm, which look for their food, these particles are proposed to keep moving with a velocity *v* in the search space, looking for the best solution. The initial swarm velocity is represented in Eq. (2)

2 $$V_{i} = \left( {V_{i1} , V_{i2} , V_{i3} , \ldots \ldots ,V_{iD} } \right)$$

Using the initial particle positions and velocities to calculate the fitness value according to a predetermined fitness function.

The genetic algorithm (GA) is inspired by the principles of Genetics and Natural Selection and is popularly used for finding optimum solutions to various problems. The first step in the GA technique is to propose a set of random populations where each individual is called a chromosome, and each chromosome consists of a fixed length of strings, where a string is called a gene^69,70^. Then, the chromosomes of the first generation (1^st^ set of population) follow several steps, including selection, cross-over and mutation to create a new population. In the selection step, a number of chromosomes of best fitness values are selected to create the next generation through mating and cross-over process. In the crossover operation, parts of two parent chromosomes are swapped as described in the Eqs. (4, 5) below:

3 $$\begin{aligned} X_{i}^{t + 1} & = \alpha \times X_{j}^{t} + \left( {1 - \alpha } \right) \times X_{i}^{t} \\ X_{j}^{t + 1} & = \alpha \times X_{i}^{t} + \left( {1 - \alpha } \right) \times X_{j}^{n} \\ \end{aligned}$$

where $X_{i}^{t + 1} , X_{j}^{t + 1}$ are two new chromosomes created at time *t* + *1,*$\alpha$ refers to the crossover variable, and *i ≠ j*. After that, the new chromosomes are subjected to a mutation process where some gene values are changed, and it is given as follows:

4 $$X_{j}^{t + 1} = X_{i}^{t} + m*rand\left( {size\left( D \right)} \right),$$

where $m$ is the mutate factor and its computed as follows:

5 $$m = 0.1*\left( {H - L} \right);$$

where $H$ and $L$ are the lower and upper boundaries. According to the PSO algorithm, the particles update their locations at every iteration based on the local and global best position according to Eq. (6).

6 $$X_{i}^{t + 1} = X_{i}^{t} + V_{i}^{t}$$

where *t* is the iteration number. The velocity is also updated at every iteration according to the Eq. (7).

7 $$V_{i}^{t + 1} = w*V_{i}^{t} + C_{1} *r_{1} *\left( {pBest_{i}^{t} - X_{i}^{t} } \right) + C_{2} *r_{2} *\left( {gBest_{i}^{t} - X_{i}^{t} } \right)$$

where *w* is inertia weight, *C*_*1*_ and *C*_*2*_ are none-negative constants controlling how the global and local best position affect the particle velocity, and *r*_*1*_*, r*_*2*_ are random constants belonging to the range [0, 1]. The fitness value is calculated at every iteration until the termination condition is reached and the best solution is found.

The fitness values are evaluated for the new generation. The process of creation of new generation and evaluation of fitness is repeated till the termination condition is met^71^.

As it was mentioned earlier that the present methodology combines their merits of PSO social thinking and GA local search ability which helps in obtaining better solution. In this methodology, the PSO performs the building solution while the GA plays as local search optimiser.

The first step in this hybrid is to propose an initial random population as in PSO and the *pBest* and *gBest* values are calculated in the first iteration. Next, the new position and velocity vectors of the PSO particles are updated and fitness values are also evaluated. Then, the new position sets are subjected to GA to represent the chromosomes sets and follow the same processes explained in the previous section (i.e. selection, crossover and mutation) to infer the best solution. The chromosomes associated with the optimum solution in GA are then sent back to PSO as an updated population. The process above is repeated until the meeting of the target fitness. One way to calculate the PSOGA fitness function is to use the root mean square error (RMSE) to determine the best and worst fits for each iteration.

### Constriction coefficient-based particle swarm optimisation with chaotic gravitational search algorithm

The Constriction Coefficient-based Particle Swarm Optimisation with Chaotic Gravitational Search Algorithm (CPSOCGSA) can be categorised as a stochastic hybrid optimisation methodology. The suggested methodology combines Particle Swarm Optimisation (PSO), which is influenced by bird flocking behaviour, with the Gravitational Search Algorithm (GSA), a technique, which is inspired by Newton’s law of universal gravity. The proposed methodology utilises the exploration and exploitation capabilities of PSO and GSA to attain the optimal result^72^.

The CPSOCGSA has been proposed to enhance the exploratory outcoms inherent in the GSA, in conjunction with the convergence possibility of the Constriction Coefficient-Based PSO. To tackle the problem of being trapped in local minima, which is usualy occurs in the classic GSA, chaotic maps are proposed as a means to address the issue. The equation that merges both aforementioned techniques is presented in Eq. (8)^72^.

8 $$v_{i}^{d} \left( {t + 1} \right) = (2/\left( {\varphi - 2 + sqrt\left( {\varphi^{2} - 4\varphi } \right)} \right)v_{i}^{d} \left( t \right) + K\varphi_{1} r_{i1} \left( {a_{i}^{d} \left( t \right) - x_{i}^{d} \left( t \right)} \right) + K\varphi_{2} r_{i2} \left( {gbest - x_{i}^{d} \left( t \right)} \right)$$

In this context, the variable $v_{i}^{d}$ represents the velocity of the particles in the swarm, $(\varphi , \varphi_{1} , \varphi_{2}$) are control parameters, *K* is the Constriction Coefficient. $a_{i}^{d}$ refers to the acceleration of the particles, and *gbest* refers to the particle system’s social capability component.

The spatial coordinates of the particles are presented by Eq. (9)^72^.

9 $$x_{i}^{d} \left( {t + 1} \right) = x_{i}^{d} \left( t \right) + v_{i}^{d} \left( {t + 1} \right)$$

The usefulness of optimisation algorithms in addressing continuous benchmark test functions is evident due to the inherent simplicity that allows agents to traverse the search space and identify feasible potential solutions within this specific category of functions. However, the accurate assessment of intelligent algorithms resides in their capacity to effectively address complex non-linear test operations, such as those encountered in engineering standards. In the aforementioned situations, algorithms are required to efficiently address complex restrictions and rigorous inequalities^72^. To calculate the CPSOCGSA fitness function, a RMSE can be used to choose the best and the worst fit for each iteration.

### Particle swarm optimisation with grey wolf optimiser

Grey Wolf Optimiser (GWO), based on their creators, is driven by the leadership sequence and hunting technique of grey wolves in nature^73^. The grey wolves can be considered as the top food chain’s consumers since they are the tertiary consumers. Regardless of gender, grey wolves are split into four groups within the leadership sequence: alpha, beta, delta, and omega^74^. After Alpha wolves, beta wolves are the best solutions, according to the GWO algorithm, followed by the delta wolves. Omega wolves are the supporters of the abovementioned wolf groups and serve as the scapegoats for the submissive wolves^74^. The top wolves are thought to be the ones doing the hunting. According to Muro et al.^75^, the procedure is carried out in three phases: the chasing phase, the pursuit phase, and the attacking phase. A mathematical model based on the abovementioned hunting phases is built. The following Eqs. (10, 11) can be adapted to model the GWO mathematically:

10 $$D = \left| {C* X_{p} \left( t \right) - X\left( t \right)} \right|,$$

11 $$X\left( {t + 1} \right) = X_{p} \left( t \right) - A * D$$

In this context, the variable *t* represents the count of instantaneous iterations. *D* represents encircling the prey. *X*_*p*_ indicates the position of the prey, whereas *X* represents the location of grey wolves. The coefficients *A* and *C* are utilised for the vectors. The coefficients *A* and *C* are computed according to the following Eqs. (12, 13):

12 $$A = a x (2 * r_{1} - 1),$$

13 $$C = 2 * r_{1}$$

The number of *a* is a linear reduction from 2 to 0 as the number of iterations decreases. The variables *r*_1_ and *r*_2_ indicate random numbers selected between (0, 1).

According to the literature, PSO provides promising results in several engineering challenges. One of the most recent projects utilising PSO featured microgrid energy scheduling^76^, and the outcomes were impressive. The exploration ability of GWO, as indicated by Şenel et al.^77^, was introduced to reduce the likelihood of swarms being drawn to a local minima. It may be deduced that the hybrid PSO-GWO is efficient in terms of its capacity for exploration as well as congregation^78^. For each iteration, to calculate the PSOGWO fitness function, a RMSE can be used to determine the best and the worst fit.

### ANN model

Most current ML applications in hydrology involve ANNs, particularly feedforward back-propagation (FFBP) learning. Accurate simulations of municipal water needs across several spatial and temporal dimensions were generated by mapping the non-linear behaviour of water data using the FFBP^79,80^.

**Fig. 2:**
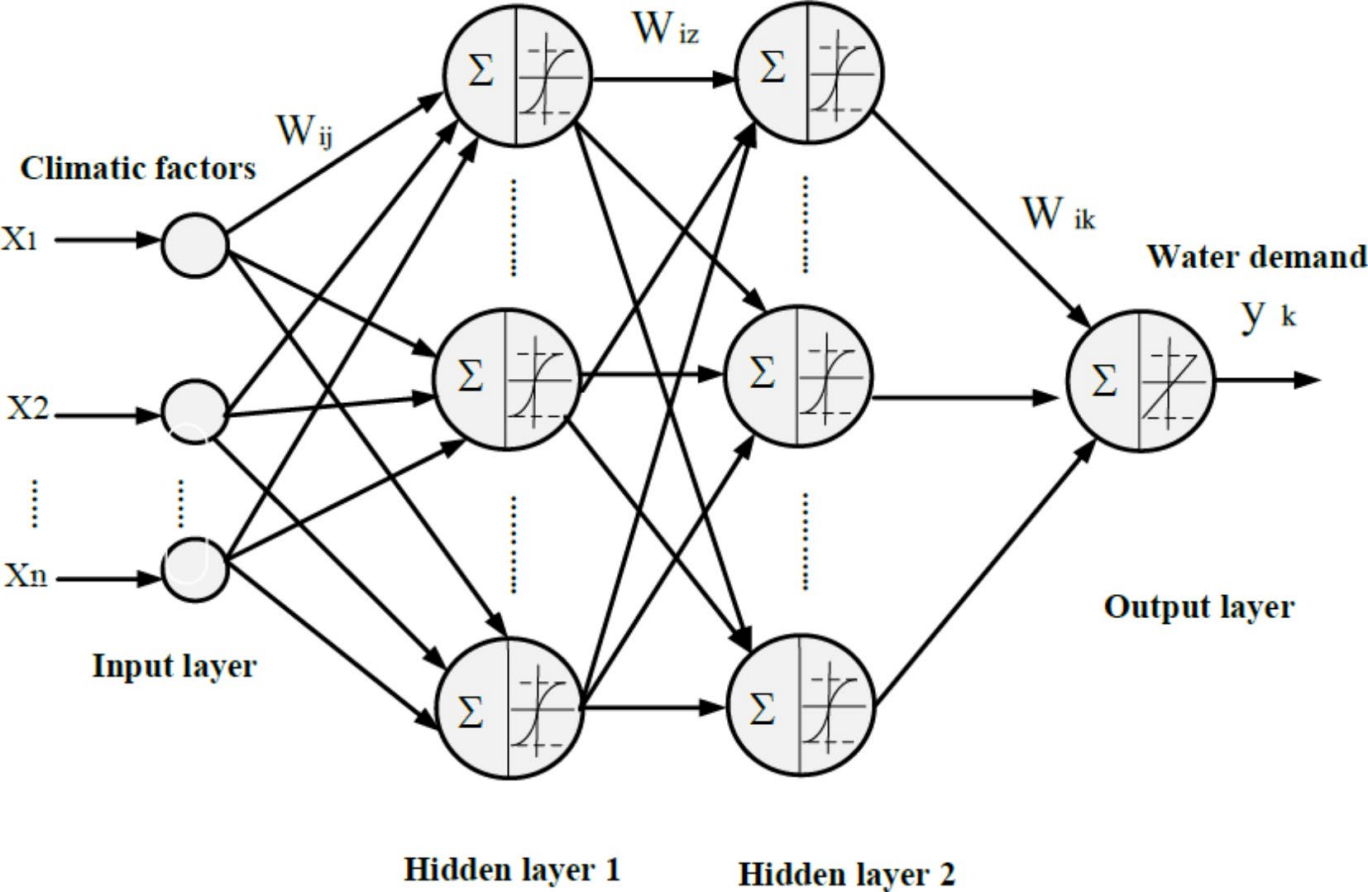
ANN model architecture.

It has been proven that with two hidden layers, ANNs can accurately simulate the non-linear relationship between predictors and targets, and this method has been effectively used by a wide range of researchers across a broad variety of applications, such as Sadeghifar et al.^81^, Shah et al.^82^, and Nunes Carvalho et al.^83^. Based on this, an ANN was constructed with four layers (Fig. 2): one for the predictors (i.e., climate factors), two hidden layers for data processing, and one for the target (i.e., urban water usage). Each of both hidden layers employs a tansigmoidal activation function, while using a linear activation function in the output layer. The dataset was divided into a training set (containing 70% of the data), a validation set (15%), and a testing set (15%), as per prior studies Zubaidi et al.^5^ and Mohammed et al.^84^. A perfect fit for ANN training is the procedure for calculating the ANN coefficients, which are the interlayer biases and weights. Consequently, optimisation approach algorithms (bayesian regularisation) are executed to improve precision^85^.

Four MHAs (i.e., PSOGA, PSOGWO, CPSOCGSA, MPSO, and PSO) were utilised separately to develop the ANN technique by finding optimum values for the ANN’s hyper-parameters (i.e., the learning rate coefficient (Lr) and the number of neurons in the first and second hidden layers (N1 and N2)).

### Validation of the model

This research used different performance criteria to assess the model performance. Due to the lack of a universally applicable performance metric, it is essential to choose the appropriate criteria for a certain application. Additionally, it is usual to utilise multiple performance criteria due to the pros and cons that exist in each criterion^86^. Also, conduct different statistical tests help to ensure the superiority of the proposed approach^87^. Several types of performance criteria used in this research include root mean squared error (RMSE, Eq. 14), mean absolute error (MAE, Eq. 15), mean absolute relative error (MARE, Eq. 16), nash sutcliffe coefficient (NSC, Eq. 17), normalised mean square error (NMSE, Eq. 18), and coefficient of determination (R^2^, Eq. 19). For a perfect model, RMSE and MAE would be zero, and NMSE, NSC, R^2^, and correlation coefficient (R) would be one^21,88,89^. Moreover, different graphical tests were used to assess the methodology, such as the Taylor diagram and Violin plot. Furthermore, four tests, including the Kolmogorov–Smirnov, Shapiro–Wilk, Augmented Dickey-Fuller (ADF), and Kwiatkowski-Phillips-Schmidt-Shin (KPSS) tests, were used to evaluate the residual data.

14 $$RMSE = \sqrt {\frac{{\mathop \sum \nolimits_{i = 1}^{N} \left( {O_{i} - F_{i} } \right)^{2} }}{N}}$$

15 $$MAE = \frac{{\mathop \sum \nolimits_{i = 1}^{N} \left| {O_{i} - F_{i} } \right|}}{N}$$

16 $$MARE = \frac{1}{N}\mathop \sum \limits_{i = 1}^{N} \frac{{\left| {O_{i} - F_{i} } \right|}}{{O_{i} }}$$

17 $${\text{NSC}} = 1 - \frac{{\mathop \sum \nolimits_{{{\text{i}} = 1}}^{{\text{N}}} \left( {{\text{O}}_{{\text{i}}} - {\text{F}}_{{\text{i}}} } \right)^{2} }}{{\mathop \sum \nolimits_{{{\text{i}} = 1}}^{{\text{N}}} \left( {{\text{O}}_{{\text{i}}} - {\overline{\text{O}}}_{{\text{i}}} } \right)^{2} }}$$

18 $$NMSE = 1 - \frac{{\mathop \sum \nolimits_{i = 1}^{N} \left| {O_{i} - F_{i} } \right|}}{{\mathop \sum \nolimits_{i = 1}^{N} \left| {O_{i} - {\overline{\text{O}}}_{{\text{i}}} } \right|}}$$

19 $$R^{2} = \left[ {\frac{{\mathop \sum \nolimits_{i = 1}^{N} \left( {O_{i} - { }\overline{O}_{i} } \right)\left( {F_{i} - F_{i} } \right)}}{{\sqrt {\sum \left( {O_{i} - \overline{O}_{i} } \right)^{2} \sum \left( {F_{i} - \overline{F}_{i} } \right)^{2} } }}} \right]^{2}$$

where $O_{i}$: observed water consumption, $F_{i}$: forecast water demand, $\overline{O}_{i}$: mean of observed water consumption, $\overline{F}_{i}$: mean of forecast water demand, N: number of data points.

## Results

### Data preprocessing techniques

Data should be preprocessed before being used to build the prediction model (as mentioned in “Data preprocessing” section). Accordingly, firstly, dependent and independents time series were normalised. Then, outliers were detected and treated.

**Fig. 3:**
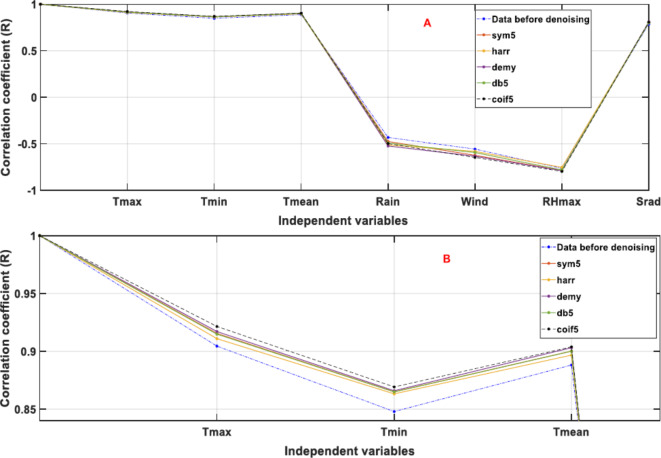
Correlation coefficients between dependent and independent factors (both raw and denoised using five distinct wavelets).

Afterwards, the WT technique was employed for denoising all the time series. Initially, sym, db, and coif wavelets were examined independently in various orders to determine the optimum order for each type. The outcomes reveal that the optimum order is 5 for sym, db, and coif wavelets. The five kinds of mother wavelets (sym5, Haar, dmey, db5, and coif5) were applied separately for denoising all-the time series, as shown in Fig. 3A, and for more details, Fig. 3B provides a clear view. The most interesting aspect of this figure is that all the types increase the model’s accuracy, and coif5 is the best.

The tolerance technique was used with different scenarios to locate the optimum predictors (weather variables) to simulate water consumption. Table 2 presents the optimum scenario of the predictors (i.e., T_max_, Rain, wind, and Rh_max_). It can be seen that the tolerance constants for all the predictors are more than 0.2, which means that there is no violation of the multicollinearity assumption.

**Table 2:** Collinearity statistics.

Predictors	Tolerance constants
T_max_	0.294
Rain	0.392
wind	0.454
RH_max_	0.272

### Models configuration

It is important to methodically design the prediction model in order to accurately estimate water demand after data has been divided into training, validation, and testing stages. For the ANN model, it is combined with a metaheuristic algorithm (PSOGA-ANN) to find the optimum hyperparameters (i.e., N_1_, N_2_, and L_r_). Other metaheuristic algorithms (i.e., PSOGWO-ANN, CPSOCGSA-ANN, and MPSO-ANN) are used to confirm the validation of the PSOGA-ANN’s results. Further, the performance of all the above hybrid models was compared with a benchmarking model (i.e., PSO-ANN) to examine to what extent the performance of the PSO algorithm was enhanced by modification or hybridised with another MHA to forecast water demand based on several weather factors.

**Fig. 4:**
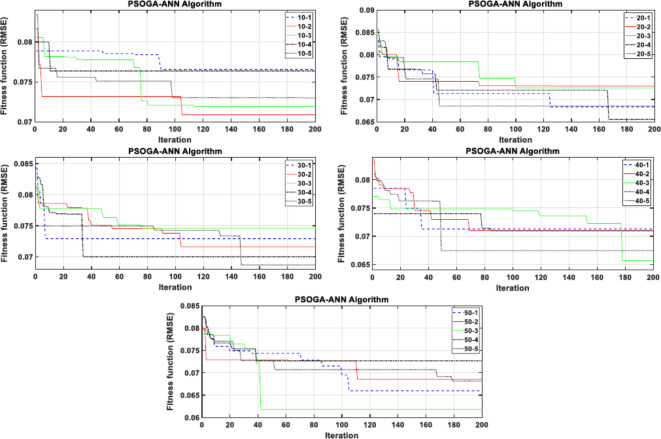
Evaluation of the PSOGA-ANN algorithm with five independent trials for different population sizes.

**Fig. 5:**
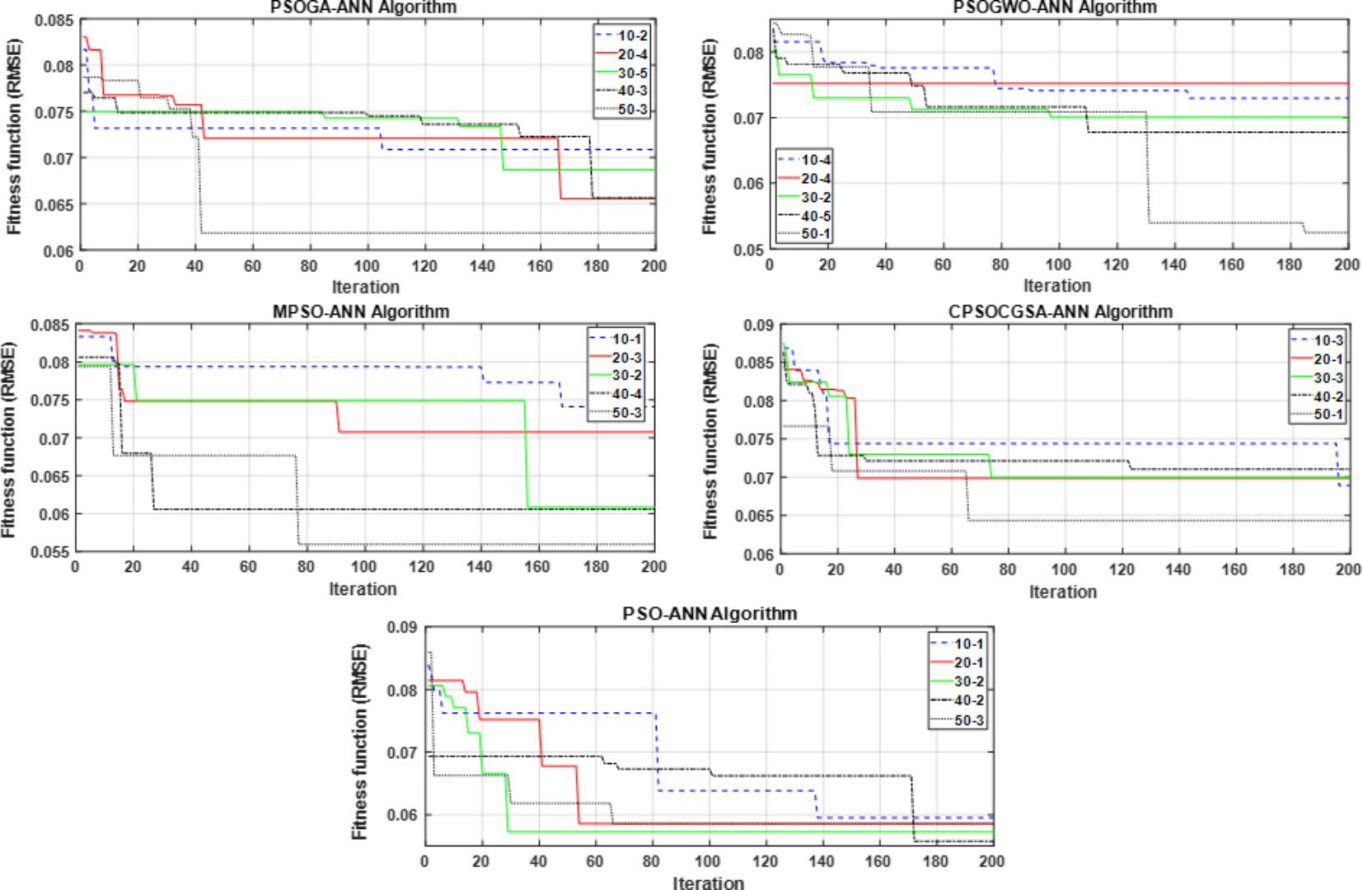
The best trial for each population size of PSOGA-ANN, PSOGWO-ANN, MPSO-ANN, CPSOCGSA-ANN, and PSO-ANN algorithms is presented.

For each algorithm, five population sizes (10, 20, 30, 40, and 50) were employed to implement the combined technique. For each population size, the forecasting range was increased, and the uncertainty was decreased by repeating the process five times (e.g., see Fig. 4 for the PSOGA-ANN algorithm). The best implementation that resulted in the lowest error (best fitness function, RMSE) was chosen for each population size (e.g., implementation two for the 10 population size of PSOGA-ANN is the best) and combined with the best implementation for the remaining population sizes (see Fig. 5 for all MHAs). Figure 5 demonstrates that the optimal population size across all methods is 50. So, the 50 population size of each hybrid model offered Lr, N1, and N2 values for their ANN model, with the exception of the PSO-ANN model, which provides the ANN model’s hyperparameters via a population size of 40.

Therefore, the ANN method for water demand simulation has been fine-tuned using the output of each MHA. Consequently, the optimal ANN hyperparameters for the best population size for each case are tabulated in Table 3.

**Table 3:** ANN-designed parameters.

Parameter	Hybrid ANN-metaheuristic models				
	PSOGA	PSOGWO	CPSOCGSA	MPSO	PSO
N1	6	1	8	1	4
N2	3	20	19	20	4
Lr	0.8194	0.9531	0.1511	1	0.2680

### Evaluation of the prediction models performance

After configuring the ANN models of prediction by locating the optimum hyperparameters for the ANN as presented in Table 3, all the models run multiple times to select the best ANN model architecture (i.e., interlayer biases and weights) to simulate the monthly water consumption. During the testing stage, various statistical criteria were applied to evaluate the methods’ capability to extrapolate urban water requests from meteorological factors.

Firstly, absolute error criteria (RMSE and MAE), maximum error criterion (Max.(error)), relative error (MARE), and dimensionless error criterion (NSC, NMSE, and R^2^) were applied for assessing and comparing technique performances. The performance assessment outcomes for the testing stage are tabulated in Table 4. The table reveals that the performance of all the proposed models is good in generalisation water demand data in accordance with Dawson et al.^88^. However, the PSOGA-ANN technique yielded lower RMSE, MAE, MARE, and Max.(error) and higher NSC, NMSE, and R^2^ than the rest of the models. These denoted that the PSOGA-ANN model represented the greatest overall performance in comparison to the other techniques.

**Table 4:** Performance assessment of four machine learning models for testing stage.

Model	Statistical criteria						
	RMSE (ML)	MAE (ML)	MARE	Max. (error) (ML)	NSC	NMSE	R^2^
PSOGA-ANN	0.06745	0.04771	0.00243	0.15245	0.929	0.939	0.947
PSOGWO-ANN	0.07492	0.05597	0.00286	0.17003	0.913	0.899	0.924
CPSOCGSA-ANN	0.07479	0.05285	0.00269	0.17936	0.913	0.904	0.923
MPSO-ANN	0.10206	0.10206	0.00438	0.21568	0.838	0.808	0.853
PSO-ANN	0.09701	0.07217	0.00367	0.19026	0.854	0.828	0.857

**Fig. 6:**
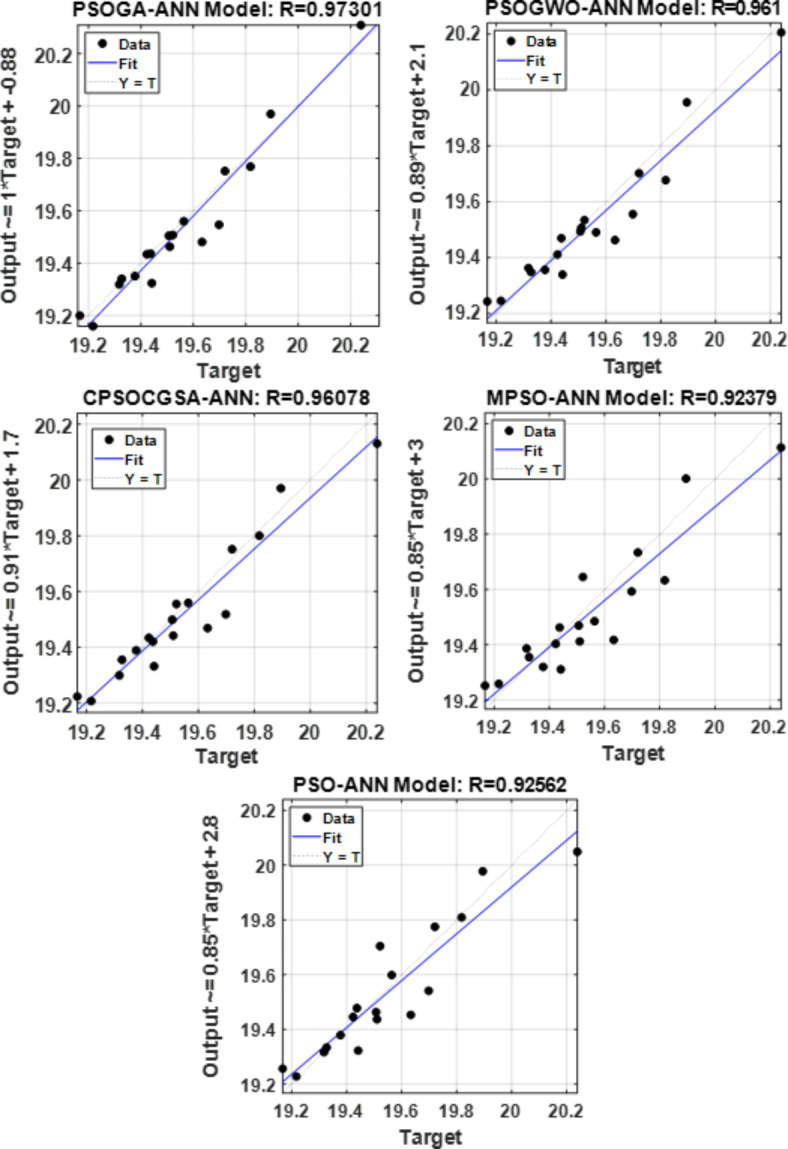
Correlation coefficient test for all proposed hybrid models.

Additional testing was conducted on the proposed models to confirm their ability to forecast College Station City’s water use. As illustrated in Fig. 6, the correlation coefficient (R) was calculated to compare the simulated and actual water consumption. The target (i.e., measured water consumption) on the x-axis is plotted versus output (i.e., forecast water request) on the y-axis. All the models offer R of more than 0.9 at the testing stage. It is equal to 0.97301, 0.961, 0.96078, 0.92379, and 0.92562 for PSOGA-ANN, PSOGWO-ANN, CPSOCGSA-ANN, MPSO-ANN, and PSO-ANN, respectively. This test confirms the capability of the PSOGA-ANN model to forecast water needs according to the criteria limitation mentioned in the section "Validation of the model." Also, it is clear that the measured and forecasted data for the PSOGA-ANN model reveal a high degree of consistency compared with other hybrid models.

**Fig. 7:**
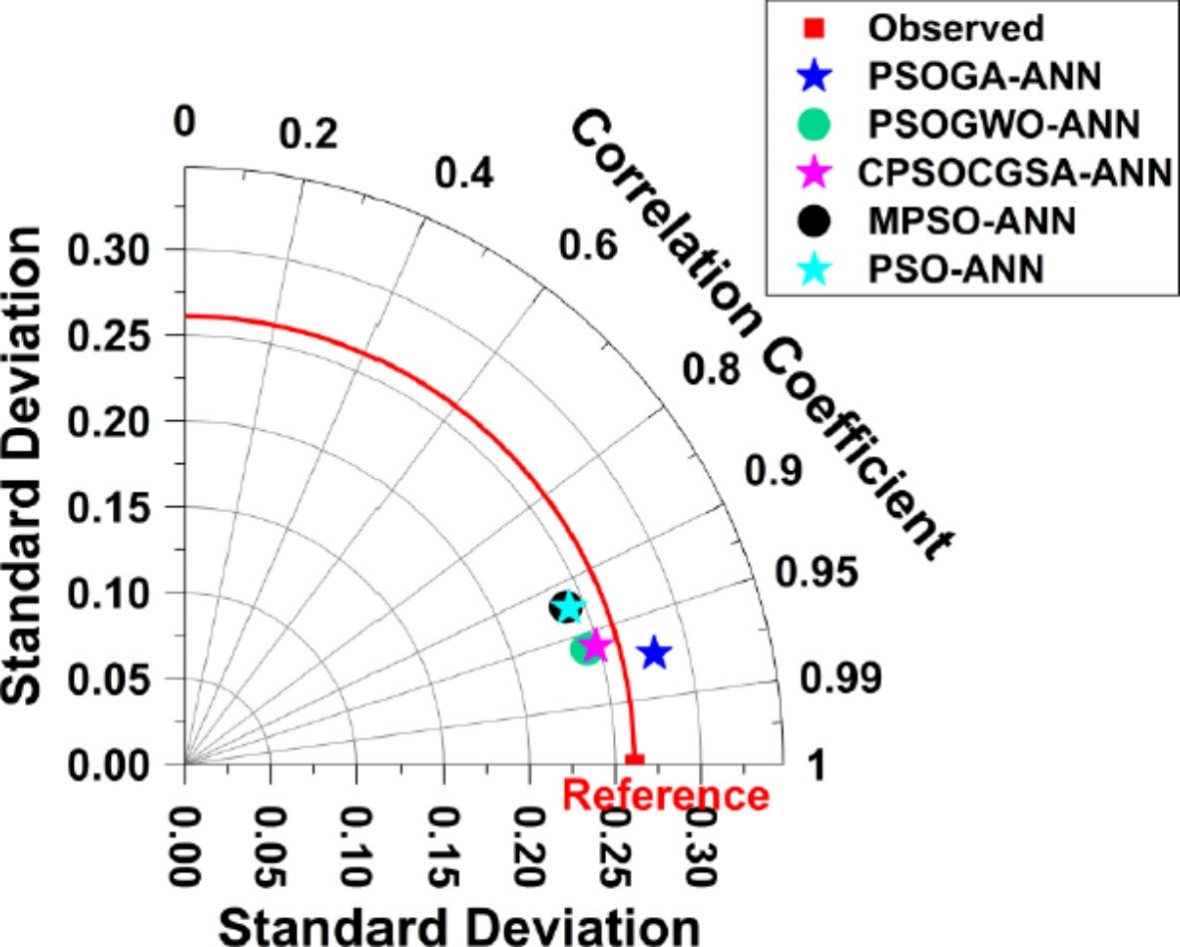
Taylor diagram for PSOGA-ANN, PSOGWO-ANN, CPSOCGSA-ANN, and MPSO-ANN techniques.

Additionally, Fig. 7 presents the Taylor diagrams that are used for preparing a visual comprehension among observed patterns (Reference) and five simulated patterns performed by PSOGA-ANN, PSOGWO-ANN, CPSOCGSA-ANN, MPSO-ANN, and PSO-ANN models. The diagram considers the root-mean-square difference (RMSD, green contour line), the standard deviation (SD, grey arc), and the correlation coefficient (R, blue azimuthal line). The diagram shows that compared to the other models (i.e. PSOGWO-ANN, CPSOCGSA-ANN, MPSO-ANN, and MPSO-ANN), the PSOGA-ANN model provided the highest R, the lowest SD and RMSD, and the nearest one from the observed pattern (Reference point).

**Fig. 8:**
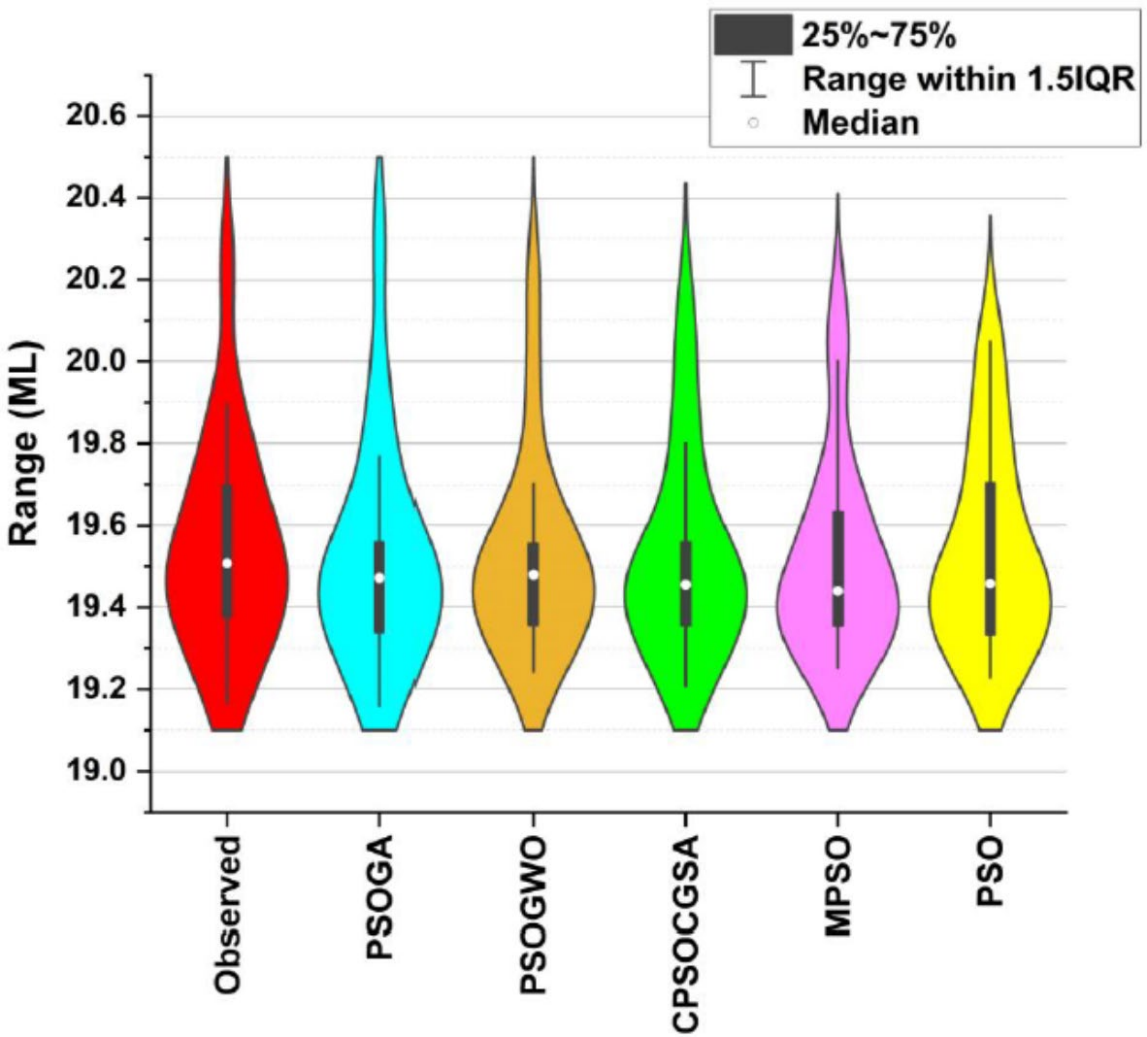
Violin plot of observed and forecasted urban water demand related to the testing stage of all suggested hybrid models.

While both violin plots and box plots are similar, the former provides more useful information. A single visualisation that integrates the box plot and density trace (or smoothed histogram) to show datasets in a complementary manner is the violin plot. Violin plots, which highlight data clusters, give a clearer picture of the distribution’s form. At each point, the violin plot indicates the amount of data that has been acquired; the top tip represents the highest data value, and the bottom tip represents the lowest data value^90^. This section utilises the violin plot diagram to compare the distribution of the measured and forecast water demand datasets during the testing period (Fig. 8). Based on the box plot limitations and violin plot distribution, the ANN-PSOGA model is more in line with the observed urban water demand than that of the other ANN-based techniques.

**Fig. 9:**
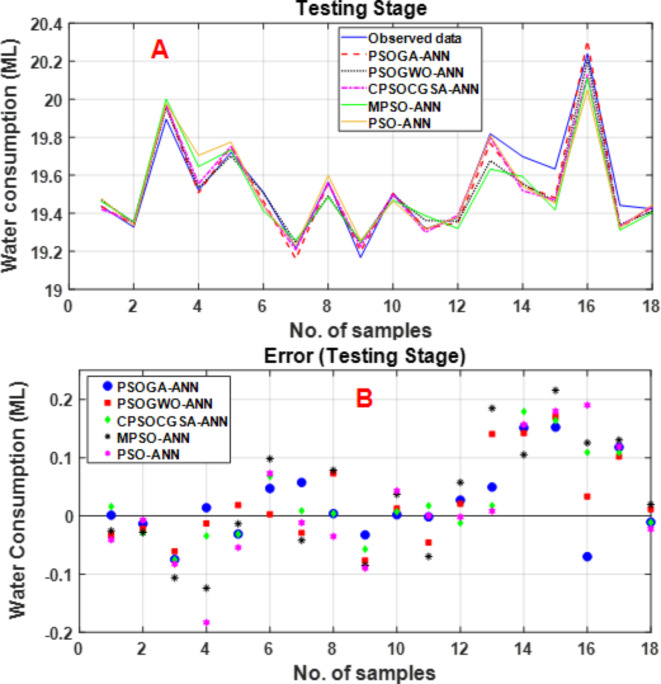
(**A**) Observed and forecasted urban water time series comparison and (**B**) Residual scatterplots for the PSOGA-ANN, PSOGWO-ANN, CPSOCGSA-ANN, MPSO-ANN, and PSO-ANN approaches in the testing phase.

Figure 9 also shows the testing stage results for the predictions of the PSOGA-ANN, PSOGWO-ANN, CPSOCGSA-ANN, MPSO-ANN and PSO-ANN models. Figure 9A presents the observed and predicted urban water time series data. In terms of pattern (trend + periodicity), the simulated time series from the PSOGA-ANN and PSOGWO-ANN models closely match the observed data. However, PSOGA-ANN performs the best, while MPSO-ANN and PSO-ANN fare the worst regarding the error scale. An error analysis was accomplished in the testing phase to examine the goodness of fit of the five hybrid models. The error scatter plots versus the sample counts for the testing phase are offered in Fig. 9B. It can be seen that relative to the other models, the PSOGA-ANN model’s error was much closer to zero (ranging from − 0.0746 to 0.1525) ML, while the rest of the error ranges are (− 0.0766 to 0.1700), (− 0.0751 to 0.1794), (− 0.1239 to 0.2157), and (− 0.1829 to 0.1903) ML for PSOGWO-ANN, CPSOCGSA-ANN, MPSO-ANN, and PSO-ANN models, respectively. The distribution also exhibited no discernible trend. According to the results shown above, PSOGA-ANN outperformed other hybrid models in terms of accuracy.

Accordingly, the PSOGA-ANN model outperformed PSOGWO-ANN, CPSOCGSA-ANN, MPSO-ANN, and PSO-ANN models over the entire range of testing data, and the MPSO-ANN model yielded the lowest performance. Finally, to increase the testing of the PSOGA-ANN model, the results of the ADF and KPSS tests indicate that the residual data points are stationary, and the normal distribution of the residuals was confirmed by the Kolmogorov–Smirnov and Shapiro–Wilk tests. Consequently, the distribution of the pattern and values of the residual data endorse the PSOGA-ANN technique’s capacity.

## Discussion

The prediction power of ML models in several branches of hydrology—including streamflow^28^, water quality^27^, and water level^26^—has been shown to be enhanced by data preprocessing and hyperparameter optimisation. Considering the limitations of data preprocessing techniques that are reported in “Intorduction” section, this paper has examined all the data preprocessing steps. The predictors and target time series underwent three preprocessing methods—normalisation (natural logarithm), a cleaning approach (WT), and choice of the optimum independents’ scenario (tolerance approach)— to maximise forecast accuracy. The WT method aids in removing random noise from the data series. Improved data quality led to higher correlation coefficients between model inputs and output, as seen in Fig. 3. A possible explanation for this result may be that particular attention is paid to normalisation and cleaning data techniques, especially the WT method, which was applied with different mother wavelets and multiple orders, resulting in better improved raw data quality.

We have also considered the consequences of selecting predictors. Accordingly, carefully choosing the predictors will also make use of all the choices in the design space that can improve the performance of the predictions and shed light on which metrological factors have the biggest impact on the output response. Thus, in order to reduce the likelihood of multicollinearity among predictors, only four of the seven climatic factors were chosen using the tolerance technique. These factors (i.e., Tmax, Rain, wind, and RHmax) have tolerance coefficients between 0.272 and 0.454, as tabulated in Table 2. These findings are in keeping with those of earlier research^82,91^, which demonstrated that reducing computation time and increasing ML model accuracy by choosing predictors using a systematic technique rather than a trial-and-error procedure.

The author´s attention was focused not only on data preprocessing but also on hybrid models of prediction. One big issue with ML techniques is their slow convergence. Another is how difficult it is to avoid local minima. Enhanced ML, thanks to recent advancements in hybrid modelling, has paved the ground for more accuracy in standalone models to be developed in the future^28^. This paper has presented several solutions to the drawbacks of hybrid models in previous studies mentioned in “Intorduction” section. This study compares the single ML model with five MHAs instead of one. Also, these MHAs are related to three inspirations instead of one. Additionally, these MHAs are three hybrid-based and two single-based algorithms. Moreover, each MHA was applied with an iteration equal to 200. Furthermore, each swarm for each MHA was implemented five times.

Since the MHAs utilised follow various strategies depending on their behaviour during the optimisation phase, the hybridisation process results in a wide range of hyperparameter values, creating multiple model scenarios. In this research, three different hybrid-based MHAs (i.e., different combinations based on their behaviour) PSOGA (SI combined with EA), CPSOCGSA (SI combined with P), and PSOGWO (SI combined with SI) were applied to locate the optimum ANN model’s hyperparameters. The performance of hybrid-based MHAs is compared with that of single-based MHA (MPSO and PSO).

According to the findings of the various statistical and graphical tests utilised to evaluate the models during the testing stage (detailed in “Results” section), it is generally accepted that both MHAs (i.e., the single-based and hybrid-based) simulated urban water demand data with good accuracy according to Dawson et al.^88^. It is probable that the improved quality of data made possible by the data preparation technique is responsible for this superiority. Additionally, the optimum solution was likely found due to running the swarm of each algorithm five times, resulting in a more robust prediction range and less uncertainty. Moreover, hybrid-based MHAs (PSOGA, PSOGWO, and CPSOCGSA) outperformed the single-based MHA (MPSO and PSO). These results confirm the findings of prior studies^92,93^ that MHAs that use a hybrid-based approach outperform those that rely on a single-based approach. However, the PSOGA-ANN algorithm is superior to generalise water demand data compared with other MHAs in the testing stage. Evidence for this can be seen in Table 4, which displays the outcomes of RMSE, MAE, MARE, NSC, NSME, and R^2^ after only a few iterations throughout the optimisation procedure (Fig. 5). Another important finding, as shown in Fig. 7, was that the hybrid-based MHAs (PSOGWO and CPSOCGSA) function similarly. Similarly, the single-based MHAs (MPSO and PSO) performance is rather close.

These results lend credence to the claim made in the previous studies^25,94^ that avoidance of local minima is possible with hybrid-based algorithms, leading to improved accuracy, stability, and reliability in solving real-world issues. In terms of future research, it would be useful to extend the current findings by examining different kinds of data preprocessing techniques. Much work remains to be done before fully understanding the extent of hybrid-based MHAs’ performance with other ML techniques is established. Also, there is a need for research studies that explore the hybrid-based MHAs’ performance with long-term datasets.

Moreover, an urban water company might theoretically utilise short-term weather forecast data to optimise the schedule of water production if they calibrate this model with their data. A water company, for instance, could lower its energy use by shifting more production to shoulder and off-peak hours if short-term weather projections called for cooler temperatures.

## Conclusion

In the last few decades, as water supplies have become scarcer and human consumption of water has rapidly grown, water utilities have focused heavily on developing more accurate methods of predicting future water needs. Motivated by data collected for College Station City, USA, over a decade, this study aimed to find a novel methodology to improve the prediction of urban water demand based on meteorological variables. This paper compared the performance of five MHAs, three of which were hybrid-based and two of which were single-based MHAs for urban water demand forecasting. The hybrid-based MHAs are PSOGA (SI combined with EA), CPSOCGSA (SI combined with P), and PSOGWO (SI combined with SI), and the single-based MHA are MPSO and PSO. These MHAs’ performance have not been compared before in terms of urban water demand. The methodology contains three combined techniques, including data preprocessing (WT and tolerance) and a prediction model (ANN that is integrated by PSOGA). The results of PSOGA-ANN were compared with those of the CPSOCGSA-ANN, PSOGWO, MPSO, and PSO algorithms.

In light of the findings, it was concluded that data pre-processing is an appropriate strategy for enhancing data quality (denoising) via the use of WT and for identifying the optimal set of predictors using tolerance. The optimum scenario of the predictors in which the multicollinearity condition is not violated was provided by Tmax, Rain, wind, and Rhmax. The performance of the PSOGA-ANN outperformed all other suggested models over the entire range of testing data stages, and the single-based models yielded the lowest performance. The PSOGA-ANN algorithm yielded R^2^, NSC, NMSE, RMSE, and MAE, of 0.947, 0.929, 0.939, 0.06745 and 0.04771 megalitres, respectively. These results indicate that the proposed methodology offers a guide to choosing appropriate predictors controlling water demand.

These conclusions have significant implications for policymakers and managers as they plan for, evaluate, and compare the accessibility of potable water supplies and growing water needs. Lastly, this research filled a gap in the literature by examining the quality and uncertainty of data analytic machine learning techniques for predicting monthly urban water demand considering weather variables. More research is needed to determine how various meteorological variables impact water demand prediction at different scales.

## Data Availability

The datasets used and/or analysed during the current study are available from the corresponding author upon reasonable request.
